# Patterns of tumor response in canine and feline cancer patients treated with electrochemotherapy: preclinical data for the standardization of this treatment in pets and humans

**DOI:** 10.1186/1479-5876-5-48

**Published:** 2007-10-02

**Authors:** Enrico P Spugnini, Feliciano Baldi, Pasquale Mellone, Florinda Feroce, Alfredo D'Avino, Francesco Bonetto, Bruno Vincenzi, Gennaro Citro, Alfonso Baldi

**Affiliations:** 1 S.A.F.U. Department, Regina Elena Cancer Institute, Rome, Italy; 2 Department of Biochemistry, section of Pathology, Second University of Naples, Italy; 3 Italian Ministry of Health, Department of Innovation, General Directorate for Medicines and Medical Device, Rome, Italy; 4 Campus Biomedico University, section of Oncology, Rome, Italy

## Abstract

Electrochemotherapy (ECT) is a novel anticancer therapy that is currently being evaluated in human and pet cancer patients. ECT associates the administration of an anti-tumor agent to the delivery of trains of appropriate waveforms. The increased uptake of chemotherapy leads to apoptotic death of the neoplasm thus resulting in prolonged local control and extended survival. In this paper we describe the histological features of a broad array of spontaneous tumors of companion animals receiving pulse-mediated chemotherapy. Multivariate statistical analysis of the percentage of necrosis and apoptosis in the tumors before and after ECT treatment, shows that only a high percentage of necrosis and apoptosis after the ECT treatment were significantly correlated with longer survivals of the patients (p < 0.0001 and p = 0.004, respectively). Further studies on this topic are warranted in companion animals with spontaneous tumors to identify new molecular targets for electrochemotherapy and to the develop new therapeutical protocols to be translated to humans.

## Background

Local management of solid neoplasms in humans generally involves multimodality approaches whose cornerstones are surgery combined with radiation therapy [1,2]. The usual radiation protocols are based on preoperative, intra-operative, or post-operative external beam treatment or adjuvant brachytherapy [3]. The aim of these strategies is to optimize local control while minimizing side effects, especially in the case of limb neoplasms. For these reasons low dose external beam fractionation is usually preferred, however in case of large cancers that involve deep underlying structures, preoperative radiation therapy might be chosen [4]. Unfortunately the rate of local wound complication associated with aggressive surgical management and radiation therapy is still elevated [5]. Electrochemotherapy (ECT) is a new approach to solid tumors that associates the administration of a chemotherapy agent to the application of square or biphasic electric pulses (EP) so to increase the uptake of drug by the cancer cells [6,7]. This approach can lead to tumor destruction through apoptotic death at least when using bleomycin as anticancer agent [8], resulting in tumor control or palliation with minimal side effects. Over the past years our group focused on the development of novel ECT protocols in pets affected by advanced cancer as a model for down-staged human patients. After preliminary studies involving also the development of custom-tailored electrodes [9,10] we studied the impact of ECT on several cohorts of canine and feline patients affected by spontaneously occurring tumors [11-13].

The high rate of local control in our preliminary investigation [9] that accounted for an overall 80% response (40% long lasting), prompted us to mount several phase II studies.

Many tumor histotypes show a marked responsiveness to pulse-mediated chemotherapy, leading to tumor shrinkage and clinical remission. In particular, ECT seems promising at controlling oral mucosal melanomas either as a single modality therapy or in conjunction with surgical cytoreduction [11]. Moreover, ECT can be employed not only to directly attack neoplasms but can also be used in an adjuvant fashion to treat residual disease, thus sterilizing the surgical field in case of incomplete excision as per radiation therapy [11-13]. Of interest, tumors reported to be resistant to this clinical strategy [14] became highly responsive when it has been coupled with surgery as evidenced by a recent publication [12].

Despite the consistent number of preclinical and clinical publications on this topic, there are few data on the histopathological modifications induced by this therapy [15]. Mir and coll. described massive necrosis induced by ECT in cats harboring post-vaccinal sarcomas, characterized by diffuse infiltration of the tumor perimeter by macrophages, lymphocytes and eosinophils [14].

The lack of extensive investigation in this field prompted us to run a thorough revision of our histological samples to gather a broader picture of patterns of tumor response and eventually to identify possible prognostic factors.

## Methods

### Electrochemotherapy protocol

**Figure 1:**
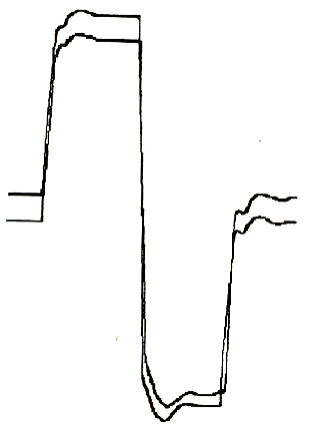
**A biphasic waveform adopted for ECT**. A diagram showing the electrical features of a biphasic electric pulse generated by a CHEMOPULSE.

A total of 127 companion animals with spontaneous tumors were enrolled in different phase II ECT trials over a 7 years period and biopsies were collected at presentation, after the first session of ECT and at the completion of the treatment (Table 1). Pets that entered in our studies received two sessions of ECT one week apart (two weeks for patients with cardiomyopathy) under sedation with medetodimine and ketamine as per manufacturer's instruction. Briefly the tumor's bed and the margins for 1/2 cm in all directions were infiltrated with bleomycin at the concentration of 1.5 mg/ml. Five minutes after the infiltration, trains of 8 biphasic electric pulses lasting 50 + 50 μs each, with 1 ms interpulse intervals, were delivered by means of modified caliper electrodes [10]. In figure 1 is shown the appearance of one of such pulses; a more thorough technical description has already been elsewhere published [7].

**Table 1:** Tumor types treated with ECT in 127 companion animals with spontaneously occurring neoplasms

**Tumor type**	**Species**	**Number of patients**
Oral melanoma	Dog	10
Soft tissue sarcoma	Cat	19 + 39
Soft tissue sarcoma	Dog	22
Mast cell tumor	Dog	28
Squamous cell carcinoma	Cat	9

### Morphological analyses

Biopsies (surgical and punch samplings) were collected before the beginning of ECT, after one session (one week after the treatment) and at the completion of the protocol (two weeks after the end of the protocol). More than 370 specimens were analyzed. Histopathology specimens embedded in paraffin have been cut into 6 μm sections and stained following standard protocols, using Hematoxylin/Eosin, Hematoxylin/Van Gieson, and toluidine blue staining (to identify poorly differentiated mast cell tumors) [16]. The TUNEL reaction was performed using the peroxidase-based Apoptag kit (Oncor, Gaithersburg, MD), as previously described [17]. In order to confirm the diagnoses, histological examination of the biopsies were independently performed by two pathologists (FB and AB) that were not informed of the clinical outcome of the veterinary patients that were staged, treated and followed up by another investigator (EPS).

### Statistical analysis

Histopathological patterns were tested for significance regarding response to first treatment, response to second session of ECT and the recurrence within the first six months of follow-up. Patients died of unrelated disease were censored in the analysis. Variables tested were: percentage of necrosis, apoptotic index. All analyses were performed following an intention to treat analysis method. The time to progression was calculated as the period from the date of starting treatment to the first observation of disease progression or to death from any cause. Survival plot were performed by Kaplan-Meier product-limit method [18]. The differences in terms of survival according to the degree of apoptosis and necrosis (both pre- and post-treatment) were evaluated by the log-rank test [19]. Finally, the Cox proportional hazards model was applied to the multivariate survival analysis [20]. SPSS software (version 11.05, SPSS, Chicago) was used for statistical analysis. A P value of less than 0.05 was considered to indicate statistical significance.

## Results

**Figure 2:**
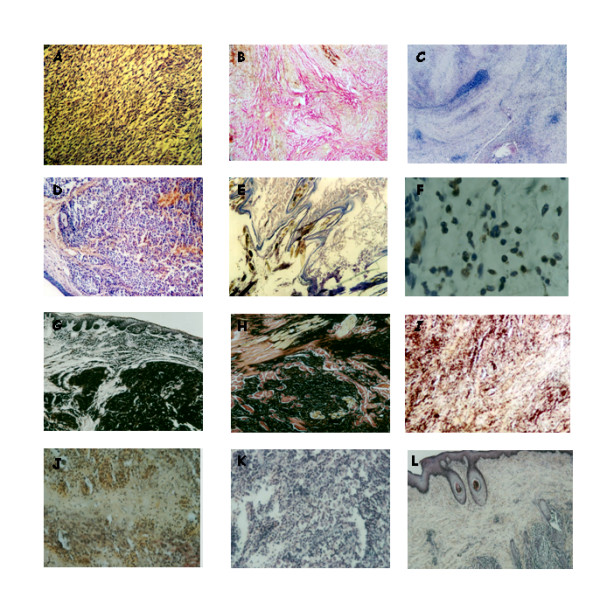
**Histopathological patterns of tumor responses to ECT treatment**. A) A high grade feline fibrosarcoma situated between the scapulae of a cat before the ECT treatment (Haematoxylin and Eosin, original magnification × 20). B) The same lesion after completion of the ECT treatment (two weeks) is shown: note the almost complete disappearance of the neoplastic cells, substituted by scar tissue (Haematoxylin/Van Gieson, original magnification × 20). C) The recurrence as a neurofibroma-like lesion at the site of a previously treated feline fibrosarcoma is depicted. The phenomenon in this particular patient occurred twelve months after the completion of the ECT course (Haematoxylin and Eosin, original magnification × 20). D) A canine cutaneous MCT before the ECT treatment (Haematoxylin and Eosin, original magnification × 20). E) The same lesion at the end of the adjuvant ECT treatment for incomplete surgical excision is shown: note the dramatic reduction in cellularity of the lesion (Haematoxylin and Eosin, original magnification × 20). F) An higher magnification of figure 1E, demonstrating that the most of the residual neoplastic cells are apoptotic (TUNEL reaction, original magnification × 40). G) A canine oral melanoma before ECT treatment (Haematoxylin and Eosin, original magnification × 20). H) A detail of figure 1G, showing the local aggressivity of the neoplasm, that invades the muscular tissue (Haematoxylin and Eosin, original magnification × 40). I) The same tumor after completion of the ECT treatment (two weeks): most of the neoplastic cells are destroyed and substituted by scar tissue (Haematoxylin/Van Gieson, original magnification × 20). J) A cutaneous squamous cell carcinoma in a cat after the first ECT treatment: note the partial destruction of the neoplasm and the inflammatory process, consisting mainly of lymphocytes, neutrophils and plasma cells (Haematoxylin and Eosin, original magnification × 20). K) A very aggressive canine haemangioperycitoma before ECT treatment (Haematoxylin and Eosin, original magnification × 40). L) The same neoplasm after two ECT treatments: note the partial destruction of the tumor, substituted by scar tissue and the integrity of the normal skin, over the neoplasm (Haematoxylin and Eosin, original magnification × 20).

**Figure 3:**
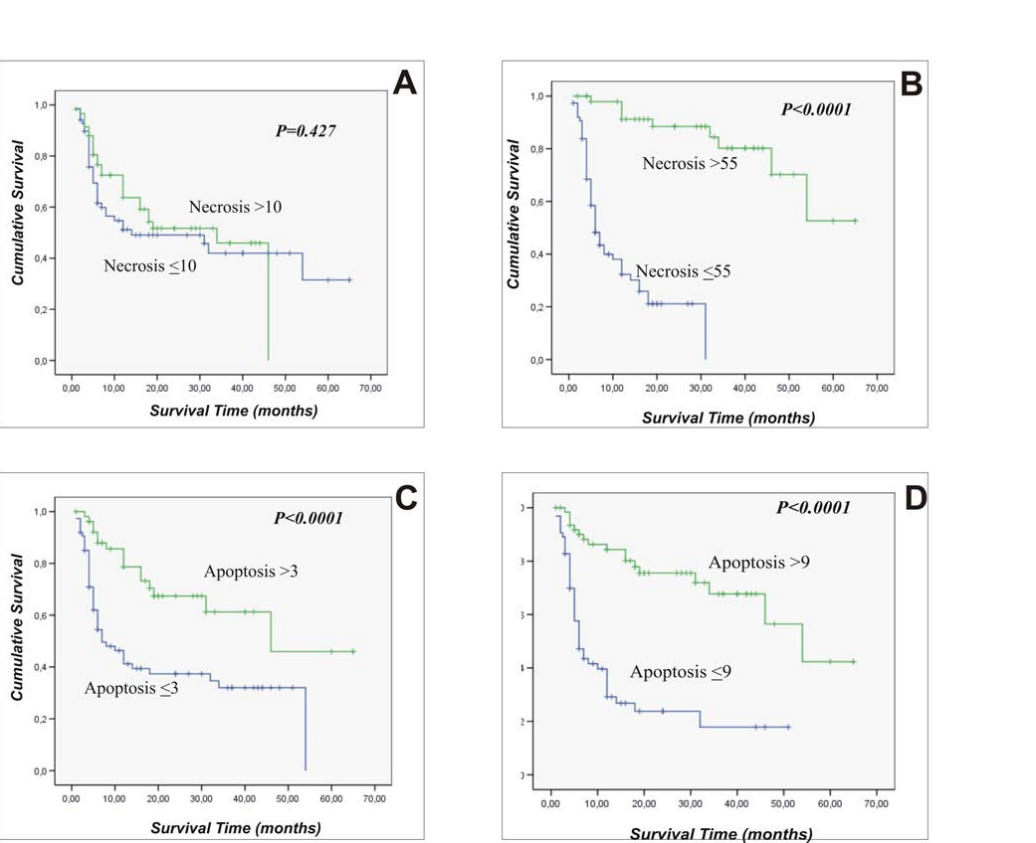
Kaplan-Meier survival plots for necrosis before ECT treatment (A); necrosis after the first ECT treatment (B); apoptosis before ECT treatment (C); apoptosis after the first ECT treatment (D).

The review of more than 370 bioptic specimens allowed to determine different features of tumor histopathology following ECT. Patterns of response in the early phases of the treatment (after 1 session of ECT) involved an acute inflammatory response made up of neutrophils, lymphocytes and plasmacells, followed by extensive necrosis. At the completion of the treatment (after two weeks), the tumor samples showed a dramatic decrease in cell number, with the majority of the remaining cells in apoptosis with no inflammatory response, while most of the residual tumor mass was made up of scar tissue. In three cases of feline sarcomas that experienced local failure, the tumor recurred as a less aggressive histotype: a neurofibroma-like lesion rather than an high grade sarcoma. Interestingly, we always detected lack of sufferance in the normal tissues surrounding the neoplasm. Table 2 summarizes the histopathological features of the ECT-treated cancers, that have been encountered in this study. In figure 2 several examples of the histopathological appearance of different tumors, are depicted (see the figure legend for the details). When we statistically analyzed the percentage of necrosis and apoptosis in the tumors before and after the first ECT treatment, we found that high percentage of necrosis after the ECT treatment was significantly correlated with survival (p < 0.0001), while the extent of necrosis in the untreated tumors did not correlate with survival (p = 0.427). On the other hand an high apoptotic rate of both untreated tumors and tumor analyzed after the first ECT treatment significantly correlated with survival (p < 0.0001). In figure 3 the corresponding Kaplan-Meier curves are depicted. Interestingly, when we performed multivariate analysis on the same histopathological parameters, we found that only high levels of necrosis and apoptosis after the ECT treatment significantly correlated with a better longer survival of the patients (Table 3).

**Table 2:** Main histopathological features of the ECT-treated cancers

Phase of treatment*	**Histopathological pattern**
Early phase (First session of ECT)	acute inflammatory response followed by necrosis and apoptosis
End of treatment (second session of ECT)	no inflammatory response, necrosis, scar tissue and apoptosis of the residual tumor cells

* See the text for the detailed description of the treatment

**Table 3:** Correlation with survival in multivariate analysis of the histopathological parameters selected

**Risk factor**	**Relative risk (95% C.I.)**	**P value**
Necrosis post > 55	0.066 (0.024 – 0.179)	<0.0001
Necrosis post ≤ 55	1	
Apoptosis pre > 3	0.534 (0.267 – 1.072)	0.078
Apoptosis pre ≤ 3	1	
Apoptosis post > 9	0.352 (0.173 – 0.715)	0.004
Apoptosis post ≤ 9	1	

Pre = before ECT treatment; Post = after the first ECT treatment

## Discussion

ECT has several advantages on other anti-tumor techniques: ease of administration, low cost, minimal toxicity (usually limited to mild focal inflammation at the treated site), and high response rate. Our morphological study shows on a broad selection of tumors of companion animals a progressive and highly selective destruction that frequently allowed conservative surgeries in the case of extensive tumors. The histopathological analysis of the treated tumor revealed that cancer cells did not undergo the typical response to bleomycin consisting with enlargement and polynucleation [8], but evidenced marked necrosis circumscribed to the tumour tissue, and an associated acute inflammatory response mediated by lymphocytes and plasma cells. In the second phase of the treatment, neoplastic apoptotic cells are detected in most of the tumour mass, with no signs of acute inflammation. This apoptotic pattern could be a consequence not only of the ECT but also a phenomenon mediated by cellular immunity, depending on the presence of a mixed T and B lymphocyte population (data not shown). When we statistically analysed the levels of necrosis and apoptosis in the tumours before and after ECT treatment, we found that either high necrosis and high apoptosis in the treated tumours were correlated with an higher survival. As it was logical to think, the percentage of necrosis in the untreated tumours did not display any significant correlation, while an high apoptotic rate in the untreated tumours was, indeed, significantly correlated with survival. These data are in agreement with several observation that in several human and animal different tumour histotypes, the apoptotic rate is an important prognostic factor [21]. Nevertheless, when we performed a multivariate analysis with the same histopathological parameters, we found that only an high level of necrosis and apoptosis after the ECT treatment, significantly correlated with a longer survival. This result reinforces the idea that it is the destruction of the tumours by the ECT treatment that is responsible for the survival of the patients.

On the other hand, this pattern of tumor lysis seems to play a key role in the prevention of local recurrence and distant dissemination for melanomas treated with this technique. In fact, we recently described a vitiligo-like lesion in canine malignant melanomas of the oral cavity where the absence of any pigment at the treatment site in the long term survivors might imply the aiming of the immune system to melanin and other deep melanoma antigens [11]. Another pattern of response, at the moment observed only in three cases of feline high grade sarcomas, suggests that ECT seems to promote a selection of tumor cells, leading to a local recurrence in the form of a less aggressive histotype: a neurofibroma-like lesion rather than an high grade sarcoma.

To the best of our knowledge, this is the most extensive description of histopathological patterns of response to electrochemotherapy in tumors of companion animals, showing previously undescribed mechanisms of response. The morphological analysis further confirm the efficacy and selectivity of this novel anticancer treatment as evidenced by the lack of sufferance elicited in the normal tissues surrounding the neoplasms. Studies are currently ongoing at our laboratory to further define the nature of the immune response elicited by the ECT treatment, in order to refine and ameliorate the efficacy of our protocols.

## Conclusion

Spontaneous tumors of pets share striking clinical, histopathological and molecular similarities with their human counterparts, thus providing an invaluable bench to clinic bridging [22].

The results of our studies are being currently translated to humans [23]. Indeed, the data presented in this article, combined with the preclinical results obtained in companion animals will be instrumental to identify new molecular targets and to plan more efficacious and less invasive ECT protocols to be transferred to humans.

## Competing interests

The author(s) declare that they have no competing interests.

## Authors' contributions

All authors read and approved the final manuscript. EPS set up the ECT protocols and treated the animals; FB, PM, FF and ADA prepared the histological samples and described the histopathological patterns; GC gave advise on the work and helped in the interpretation of the data; FB and BV performed the statistical analysis; AB supervised all the work and wrote the paper together with EPS.
